# Ivermectin susceptibility, sporontocidal effect, and inhibition of time to re-feed in the Amazonian malaria vector *Anopheles darlingi*

**DOI:** 10.1186/s12936-017-2125-0

**Published:** 2017-11-21

**Authors:** Kevin C. Kobylinski, Karín S. Escobedo-Vargas, Victor M. López-Sifuentes, Salomón Durand, Edward S. Smith, G. Christian Baldeviano, Robert V. Gerbasi, Sara-Blythe Ballard, Craig A. Stoops, Gissella M. Vásquez

**Affiliations:** 1 Department of Entomology, Armed Forces Research Institute of Medical Sciences, 315/6 Rajvithi Road, Bangkok, 10400 Thailand; 2 Entomology Branch, Walter Reed Army Institute of Research, 503 Robert Grant Ave, Silver Spring, MD 20910 USA; 3 Department of Entomology, U.S. Naval Medical Research Unit No. 6, Av. Venezuela block 36 s/n, Callao 2, Peru; 4 Department of Parasitology, U.S. Naval Medical Research Unit No. 6, Av. Venezuela block 36 s/n, Callao 2, Peru; 5 Infectious Diseases Directorate, Naval Medical Research Center, Silver Spring, MD 20910 USA; 6 Department of International Health, Johns Hopkins Bloomberg School of Public Health, 615 N. Wolfe St., Rm. W5515, Baltimore, MD 21205 USA

**Keywords:** Ivermectin, *Anopheles darlingi*, *Plasmodium vivax*, Amazon

## Abstract

**Background:**

Outdoor malaria transmission hinders malaria elimination efforts in the Amazon region and novel vector control tools are needed. Ivermectin mass drug administration (MDA) to humans kills wild *Anopheles*, targets outdoor-feeding vectors, and can suppress malaria parasite transmission. Laboratory investigations were performed to determine ivermectin susceptibility, sporontocidal effect and inhibition of time to re-feed for the primary Amazonian malaria vector, *Anopheles darlingi*.

**Methods:**

To assess ivermectin susceptibility, various concentrations of ivermectin were mixed in human blood and fed to *An. darlingi*. Mosquito survival was monitored daily for 7 days and a non-linear mixed effects model with Probit analysis was used to calculate lethal concentrations of ivermectin that killed 50% (LC_50_), 25% (LC_25_) and 5% (LC_5_) of mosquitoes. To examine ivermectin sporonticidal effect, *Plasmodium vivax* blood samples were collected from malaria patients and offered to mosquitoes without or with ivermectin at the LC_50_, LC_25_ or LC_5_. To assess ivermectin inhibition of mosquito time to re-feed, concentrations of ivermectin predicted to occur after a single oral dose of 200 μg/kg ivermectin were fed to *An. darlingi*. Every day for 12 days thereafter, individual mosquitoes were given the opportunity to re-feed on a volunteer. Any mosquitoes that re-blood fed or died were removed from the study.

**Results:**

Ivermectin significantly reduced *An. darlingi* survivorship: 7-day-LC_50_ = 43.2 ng/ml [37.5, 48.6], -LC_25_ = 27.8 ng/ml [20.4, 32.9] and -LC_5_ = 14.8 ng/ml [7.9, 20.2]. Ivermectin compound was sporontocidal to *P. vivax* in *An. darlingi* at the LC_50_ and LC_25_ concentrations reducing prevalence by 22.6 and 17.1%, respectively, but not at the LC_5_. Oocyst intensity was not altered at any concentration. Ivermectin significantly delayed time to re-feed at the 4-h (48.7 ng/ml) and 12-h (26.9 ng/ml) concentrations but not 36-h (10.6 ng/ml) or 60-h (6.3 ng/ml).

**Conclusions:**

Ivermectin is lethal to *An. darlingi*, modestly inhibits sporogony of *P. vivax*, and delays time to re-feed at concentrations found in humans up to 12 h post drug ingestion. The LC_50_ value suggests that a higher than standard dose (400-μg/kg) is necessary to target *An. darlingi*. These results suggest that ivermectin MDA has potential in the Amazon region to aid malaria elimination efforts.

## Background

Malaria incidence is declining globally and rates are falling in South America. Increased malaria control measures and access to effective artemisinin combination therapy for *Plasmodium falciparum* are attributed to this reduction, with *Plasmodium vivax* now being more prevalent than *P. falciparum* in the Americas, including the Amazon region [1, 2]. *Anopheles darlingi* is considered the primary malaria vector in the Amazon region [3]. Vector control is complicated by the exophagic and exophilic tendencies of *An. darlingi* combined with the fact that it typically occurs in recently cleared areas of the jungle where people may have limited access to vector control measures and health care [4–8]. Therefore, developing novel vector control strategies which can target exophagic and exophilic malaria vectors such as *An. darlingi* is essential to malaria elimination efforts in South America.

Numerous laboratory studies [9, 10], animal studies, and clinical trials [11, 12] have demonstrated that ivermectin is lethal to more than a dozen species of *Anopheles* worldwide. Ivermectin mass drug administration (MDA) has been suggested as a possible malaria parasite transmission control tool as it directly targets the vector at the point of human blood feeding, making it one of the few vector control tools under investigation that can directly target outdoor malaria transmission. Ivermectin MDAs in West Africa [13, 14] and the South Pacific [15, 16] validated that ivermectin is lethal to wild *Anopheles* at human-relevant concentrations. Furthermore, the ivermectin MDAs in Senegal, Liberia and Burkina Faso demonstrated that ivermectin can suppress *P. falciparum* transmission by wild *Anopheles gambiae* s.l. [14, 17]. In addition to mosquito-lethal effects, ivermectin at mosquito-sub-lethal concentrations affects additional parameters of vectorial capacity by inhibiting *Plasmodium* development in the vector [9, 18, 19] and delaying mosquito time to re-feed [20].

Ivermectin MDA campaigns have been performed in Central and South America for onchocerciasis elimination efforts by the Onchocerciasis Elimination Programme for the Americas (OEPA). These MDAs have been very effective at reducing *Onchocerca volvulus* transmission and have now eliminated the parasite from 11 of the 13 original foci [21–25]. Initially ivermectin MDAs for onchocerciasis were performed once or twice per year in Latin America. Later, it was determined that ivermectin could be given safely every 3 months [26] with quarterly MDAs effectively reducing transmission burden when nearing elimination [27]. Policy was changed and ivermectin MDAs have been deployed up to four times annually at various onchocerciasis elimination foci in Latin America [21]. This illustrates that frequent ivermectin MDAs with effective population coverage can be orchestrated in Latin America, and suggests that the more frequent ivermectin MDAs required to suppress malaria transmission [28] could be possible.

Before ivermectin MDAs for malaria parasite transmission suppression can be implemented in Latin America, the effects of ivermectin on key malaria vectors in the region, such as *An. darlingi*, must be evaluated. Laboratory studies were conducted to investigate the effect of ivermectin compound on *An. darlingi* survivorship, *P. vivax* development in *An. darlingi* and whether ivermectin delays the *An. darlingi* time to re-feed.

## Methods

### Mosquitoes

All *An. darlingi* were reared at the Naval Medical Research Unit No. 6 (NAMRU-6) in Iquitos, Peru as described previously [29]. Larvae were raised in the larvae insectary room (26.8 ± 0.7 °C and 76.1 ± 6.3% relative humidity, and 12-h light:12-h dark photoperiod) and adults were maintained in the adult insectary room (25.9 ± 0.8 °C and 69.7 ± 5.7% relative humidity, and 12-h light:12-h dark photoperiod). Adult mosquitoes used for experiments were provided with 10% sucrose solution ad libitum. Mosquitoes used for experiments were between 3 and 5 days post emergence and mosquitoes were sugar starved with access to water from 18 to 22 h prior to their first blood meal.

### Drug

Ivermectin was prepared as described previously [9]. Powdered formulation of ivermectin compound was obtained from Sigma-Aldrich (St. Louis, MO, USA). Ivermectin was dissolved in dimethylsulfoxide (DMSO) to concentrations of 10 mg/ml and 20 µl aliquots were frozen at – 20 °C. Ivermectin was thawed and serial dilutions were made in phosphate buffered saline (PBS) and 10 μl was added to 990 μl of blood to reach final concentration desired for mosquito membrane-feeding assays. Control blood meals consisted of previously frozen DMSO diluted in PBS to match the ratio of DMSO and PBS fed to mosquitoes in the ivermectin-containing blood meals.

### Blood

Chicken blood to maintain the mosquito colonies was obtained from a local slaughter-house consistent with previous studies [29]. Blood for lethal concentration calculation experiments were drawn from healthy volunteers and malaria-infected patients into 10 ml sodium heparin tubes (NH) (158 USP units, BD Vacutainer, Franklin Lakes, NJ, USA). Blood for lethal concentration experiments was stored at 4 °C and never more than 2 weeks post collection at the time of mosquito blood feeds. *Plasmodium vivax*-infected patients were identified by microscopic examination of Giemsa-stained thick blood smears at Ministry of Health health centres and hospitals in Iquitos. Patients were transported to NAMRU-6, enrolled and venous blood (15-ml) was drawn on site for the ivermectin sporogony experiments following procedures approved by NAMRU-6 and Walter Reed Army Institute of Research Institutional Review Boards (NMRCD.2008.0004 and WRAIR#2175) in compliance with all applicable Federal regulations governing the protection of human subjects. Blood for the ivermectin re-feeding experiments was collected from four healthy, non-malarious volunteers, as determined by microscopy and confirmed by a pre-optimized nested PCR assay [30, 31] (NAMRU6.2014.0009 and WRAIR#2342).

### Ivermectin lethal concentration calculations

Lethal concentration calculations for *An. darlingi* were performed as described previously [9]. *Anopheles darlingi* were blood fed multiple concentrations of ivermectin to determine the lethal concentration that killed 50% (LC_50_), 25% (LC_25_) and 5% (LC_5_) of the mosquitoes following previous methods [18, 20]. Control blood meals consisted of DMSO diluted in PBS to match the concentration found in the highest ivermectin treatment group in each replicate. After blood feeding via a membrane feeder, blood-fed mosquitoes were gently aspirated from the feeding container and transferred to clean, 250-ml, cardboard containers with access to 10% sucrose and kept in the infection insectary room at 24.8 ± 1.0 °C and 62.1 ± 6.7% relative humidity, on a 12-h light:12-h dark photoperiod. Mosquito survivorship was monitored for 7 days, every 24 h dead mosquitoes were removed and recorded and on day 7 all remaining mosquitoes were frozen and counted as alive.

### Effect of ivermectin on *Plasmodium vivax* sporogony

*Plasmodium vivax*-infected blood was collected from malaria-infected patients as described above. Ivermectin at LC_50_, LC_25_ and LC_5_ concentrations and pair-matched DMSO controls were added to 1 ml of whole blood and an additional 1 ml of whole blood without ivermectin or DMSO control were fed to approximately 100 *An. darlingi* per 2.5-l plastic container. Unfed mosquitoes were removed from the container and discarded. Blood-fed mosquitoes were left in the container and provided with 10% sucrose solution. Mosquitoes infected with *P. vivax* were securely maintained in the infection insectary room.

Mosquitoes were dissected 7 days post parasite ingestion to enumerate oocysts. Midguts were dissected with minuten pins into saline on a microscope slide and stained with 0.1% mercurochrome and viewed at 40× magnification with a compound microscope to determine oocyst prevalence and intensity. Approximately 25 mosquitoes were dissected from each control and treatment group.

### Ivermectin inhibition of time to re-feed

Four ivermectin concentrations predicted to occur at 4, 12, 36, and 60 h post ingestion of the 200 µg/kg dose [32] were determined as described previously [20]. Blood was drawn from the healthy volunteers and mixed with the four ivermectin concentrations and a DMSO control matched to the highest ivermectin concentration. One ml of blood for each concentration was offered to 100 *An. darlingi* females via a membrane feeder. Twenty fully engorged females from each concentration were individually transferred to separate 50-ml conical tubes. Each tube had cotton padding and filter paper placed on the bottom of the tube and moistened with 5 ml of distilled water, and the top of the tube was sealed with mesh netting. Mosquitoes were maintained in the adult insectary room and held without access to sugar for the remainder of the experiment. Every 24 h the volunteers returned to NAMRU-6 to re-feed the mosquitoes. Volunteers laid their forearms across the tops of the 50-ml tubes for 5 min. Care was taken to ensure that volunteers blood fed only the mosquitoes that ingested their original blood samples. Once all the mosquitoes had been given the opportunity to re-feed they were investigated visually for blood meal ingestion or death by shining a bright headlamp onto the mosquito. Any blood fed or dead mosquitoes were removed from the experiment and recorded. Volunteers returned to NAMRU-6 for 12 consecutive days for mosquito re-feeding opportunities. Any mosquitoes alive at the end of the experiment were recorded (NAMRU6.2014.0009 and WRAIR#2342).

### Statistical analysis

Mosquito survival and sporontocidal results were analyzed as described previously [9]. A non-linear mixed effects model with Probit analysis was used to calculate 7-day-LC_50_, -LC_25_ and -LC_5_ values with Statistical Analysis Software (SAS Institute, Inc., Cary, NC, USA) [20]. Hazard ratios for mosquito mortality at day 7 post-blood meal were calculated using Poisson regression analysis with STATA version 12.1 (Stata Corp, LLC, College Station, TX, USA). Oocyst prevalence (i.e., proportion of infected mosquitoes) was compared by Fishers Exact test. Oocyst intensity (i.e., number of oocysts per infected mosquito) was compared by the Mann–Whitney U test.

In the re-feeding experiment, mosquitoes that died instead of re-blood feeding, or survived to the end of the 12 days were censored data (up-ticks marked on each graph line). Replicates were pooled and analysed by the Logrank Test (Mantel–Cox method; proportional hazards model) and the hazard ratio with 95% confidence intervals. The Fisher’s Exact, Mann–Whitney U, and Logrank test analyses were performed with Prism 7 (GraphPad Software, Inc, San Diego, CA, USA).

## Results

### Ivermectin lethal concentration calculations

A total of 6161 mosquitoes and 13 replicates were used to calculate the lethal concentration of ivermectin. The *An. darlingi* ivermectin lethal concentrations and 95% fiducial limits were estimated at day 7 as: LC_50_ = 43.2 ng/ml [37.5, 48.6], LC_25_ = 27.8 ng/ml [20.4, 32.9], and LC_5_ = 14.8 ng/ml [7.9, 20.2]. All ivermectin concentrations had significantly increased hazard of mortality compared to the control group except for 15, 12, 10, 8, and 4 ng/ml, while 12, 8 and 4 ng/ml had significantly reduced hazard for mortality (Table 1).

**Table 1:** Hazard of mosquito mortality post ivermectin blood meal

Conc.	IRR	[95% CI]	P value
70	5.7	[4.2–7.7]	*<* *0.0001*
65	5.0	[3.8–6.4]	*<* *0.0001*
60	4.7	[3.6–6.1]	*<* *0.0001*
50	4.1	[3.3–5.0]	*<* *0.0001*
45	2.6	[2.0–3.4]	*<* *0.0001*
40	3.0	[2.4–3.7]	*<* *0.0001*
35	2.1	[1.6–2.7]	*<* *0.0001*
30	2.4	[1.9–3.1]	*<* *0.0001*
25	2.0	[1.4–2.8]	*<* *0.0001*
20	1.6	[1.3–2.1]	*<* *0.0001*
15	1.2	[0.9–1.6]	0.27
12	0.5	[0.3–0.9]	*0.018*
10	0.9	[0.6–1.3]	0.567
8	0.4	[0.2–0.7]	*0.004*
4	0.4	[0.2–0.7]	*0.002*

*Conc.* concentration of ivermectin imbibed in ng/ml, *IRR* incidence rate ratio of mortality at day 7 between each treatment group divided by the control group, *95% CI* 95% confidence intervals

Significant P values (P < 0.05) are in italic

### Effect of ivermectin on *Plasmodium vivax* sporogony

When ivermectin and *P. vivax* were co-ingested *by An. darlingi* it reduced oocyst prevalence at the ivermectin LC_50_ by 22.6% (χ^2^ = 10.32, *P* = 0.0014, reps = 7, n = 287) and LC_25_ by 17.1% (χ^2^ = 5.16, *P* = 0.0314, reps = 7, n = 285), and increased oocyst prevalence but not significantly at the LC_5_ by 11.3% (χ^2^ = 1.95, *P* = 0.1918, reps = 7, n = 283) (Fig. 2). Mean oocyst intensity was reduced slightly at the LC_50_ by 2.3% (*P* = 0.6914, reps = 7, n = 196), increased at the LC_25_ by 37.3% (*P* = 0.1838, reps = 7, n = 194), and was reduced slightly at the LC_5_ by 4.6% (*P* = 0.8595, reps = 7, n = 200) (Fig. 3) but none of these trends was significant.

### Ivermectin inhibition of time to re-feed

The ivermectin concentrations used for the re-feeding experiment were estimated from a previous clinical trial [32] at 4-h = 48.7 ng/ml, 12-h = 26.9 ng/ml, 36-h = 10.6 ng/ml, and 60-h = 6.3 ng/ml. The time to re-feed was delayed in *An. darlingi* that ingested the 4-h (48.7 ng/ml) (χ^2^ = 10.11, *P* = 0.0015, HR = 2.961 [1.631–5.377], n = 287) and 12-h (26.9 ng/ml) (χ^2^ = 6.072, *P* = 0.0137, HR = 1.987 [1.154–3.422], n = 151), but was not delayed following ingestion of 36-h (10.6 ng/ml) (χ^2^ = 0.469, *P* = 0.4935, HR = 1.043 [0.646–1.684], n = 156), nor 60-h (6.3 ng/ml) (χ^2^ = 1.502, *P* = 0.2203, HR = 1.355 [0.817–2.248], n = 145) (Fig. 4).

## Discussion

**Fig. 1:**
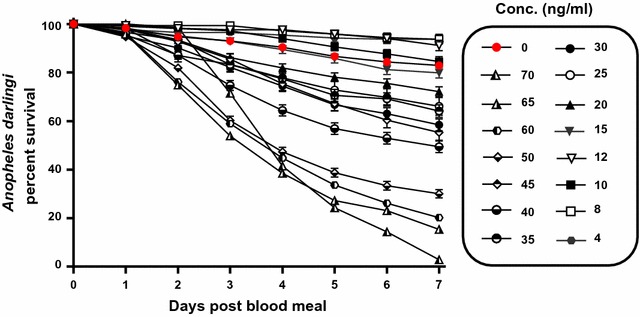
*Anopheles darlingi* survival post ingestion of ivermectin by day. Boxed legends represent the concentrations of ivermectin imbibed. Not all concentrations included in the lethal concentration analyses are displayed here. Each line represents 1–13 replicates with standard error

These findings indicate that the primary Amazonian malaria vector, *An. darlingi*, is susceptible to ivermectin compound at human-relevant concentrations (Fig. 1). The *An. darlingi* ivermectin 7-day-LC_50_ = 43.2 ng/ml is roughly equal to another South American malaria vector, *Anopheles aquasalis* 5-day-LC_50_ = 47.0 ng/ml [10]. This demonstrates that ivermectin can alter the most influential variable for vectorial capacity, the daily probability of adult survivorship [33], in two important malaria vectors in South America. Recent ivermectin pharmacokinetic modelling [9] suggests that the 400-µg/kg dose may be the ideal minimal MDA dose to target both *An. darlingi* and *An. aquasalis* in South America. Ivermectin at the 400-µg/kg dose is now recommended in some instances for lymphatic filariasis MDAs [34], and repeated doses every 2 weeks for 12 weeks were shown to be well tolerated in a trial in Brazil [35] and Sri Lanka [36].

**Fig. 2:**
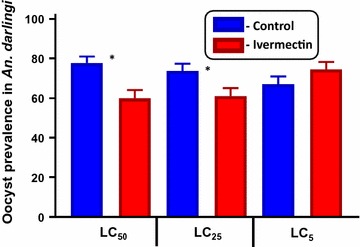
*Plasmodium vivax* oocyst prevalence in *Anopheles darlingi* when ivermectin LC_50_, LC_25_ and LC_5_ co-ingested with parasites. Oocyst prevalence was significantly reduced at the LC_50_ and LC_25_ but not the LC_5_ concentrations as determined by Fishers Exact test

**Fig. 3:**
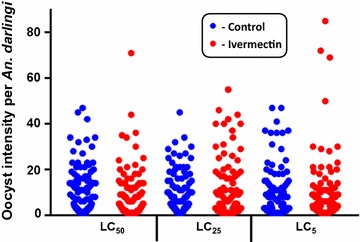
*Plasmodium vivax* oocyst intensity in *Anopheles darlingi* when ivermectin LC_50_, LC_25_ and LC_5_ co-ingested with parasites. Oocyst intensity was not reduced at any concentration as determined by Mann–Whitney U test

The sporontocidal effect of ivermectin compound against *P. vivax* in *An. darlingi* significantly, albeit modestly, reduced oocyst prevalence at the LC_50_ and LC_25_ but not the LC_5_ (Fig. 2) and did not reduce oocyst intensity at any concentration (Fig. 3). This is surprising when compared to recent sporontocidal results of ivermectin against *P. vivax* oocyst prevalence and intensity reductions at the LC_25_ and LC_5_ in *Anopheles dirus* and *Anopheles minimus* [9]. Serum replacement from malaria naïve donors was not performed in the current study with *An. darlingi*, which may explain some of the differences in ivermectin sporontocidal effect observed between studies with *P. vivax*. Previously, ivermectin LC_25_ was shown to be sporontocidal against cultured *P. falciparum* NF54 in *An. gambiae*, reducing oocyst prevalence but not intensity [18, 19]. Differences in ivermectin sporontocidal effect may be partially explained by differences in vector biology and physiology as *An. darlingi* belongs to the New World *Nyssorhynchus* subgenus while *An. dirus*, *An. minimus*, and *An. gambiae* belong to the *Cellia* subgenus. There is a shorter co-evolutionary history between *P. vivax* and New World *Anopheles*, when the parasite was possibly introduced from southern Asia in pre-Columbian times or from Europe in post-Columbian times [37]. It may be that these different vectors have different rates of peritrophic matrix formation which can be impacted by ivermectin [18] or different microbiota present in the colonized mosquitoes which could possibly be affected by ivermectin and in turn alter *Plasmodium* infection outcomes [9]. Due to this limited sporontocidal effect of ivermectin compound in *An. darlingi* at point of parasite co-ingestion, effects of ivermectin ingested at different time points from parasites were not investigated.

**Fig. 4:**
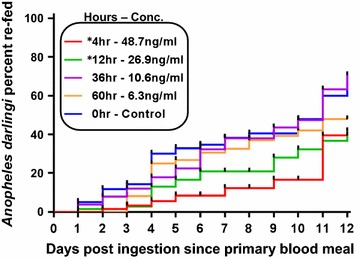
*Anopheles darlingi* time to re-feed following ingestion of ivermectin. Time to re-feed was significantly delayed at the 4-h (48.7 ng/ml) and 12-h (26.9 ng/ml) but not the 36-h (10.6 ng/ml) or 60-h (6.3 ng/ml) concentrations as determined by Logrank Test (Mantel–Cox method)

Ivermectin significantly delayed the time to re-feed for *An. darlingi* at the 4- and 12-h concentrations but not at the 36- or 60-h concentrations (Fig. 4). The time to re-feed is the second-most important variable in vectorial capacity, thus any delay at sub-lethal concentrations has ability to suppress transmission in the field [33]. Furthermore, a delay in *An. darlingi* time to re-feed may decrease the likelihood of survival which would compound mortality and further suppress *Plasmodium* transmission in the Amazon. A similar delay in time to re-feed after ivermectin ingestion was observed for *An. gambiae* [20], which may be caused by the ivermectin knockdown and delay in recovery effects also observed in *An. gambiae* [38].

The mechanism of action for ivermectin to delay *Anopheles* time to re-feed has not been characterized. Interestingly, it was demonstrated in the dung beetle, *Scarabaeus cicatricosus*, that ivermectin ingestion in dung diet decreased olfactory response and locomotor function, suggesting a negative impact on insect basic biological activities, such as food or mate-seeking [39]. In *An. gambiae*, the glutamate-gated chloride ion channel, which is the target of ivermectin, was found in the thoracic ganglia, Johnston’s organ, antennal segments, optic lobe and supraesophageal ganglion [40]. The thoracic ganglia contain motor neurons for regulating locomotor function of flight and leg muscles while the Johnston’s organ regulates flight coordination, which may explain the paralytic effect frequently observed following ivermectin ingestion by mosquitoes [38, 40]. The antenna, optic lobe and supraesophageal ganglion work in concert to regulate chemosensory and visual cues for host location, thus ingestion of ivermectin may impair the ability of mosquitoes to locate their host [40]. Moreover, reduced olfactory sensitivity towards ivermectin-treated animal host cues was recorded in the midge *Culicoides imicola* [41], further suggesting that ivermectin could inhibit host attraction and biting in haematophagous insects. Electroantennogram studies characterizing electrophysiological responses to host odour stimuli have not been performed in *Anopheles* that have ingested ivermectin. If insect olfactory detection mechanisms or other sensory capacities are impacted by ivermectin, then this will inhibit the ability of *Anopheles* to detect vertebrates when host-seeking, which could delay time to re-feed.

Ivermectin MDA campaigns conducted by OEPA demonstrate that repeated MDAs in Latin America are feasible at up to 3 month intervals. While the remoteness of some villages in the Amazon will make them difficult to contact, the successes being noted with ivermectin MDA in Yanomami indigenous populations in the Venezuelan Amazon are quite laudable [42]. Many people in areas of Latin America afflicted with malaria will likely have concomitant infections with numerous neglected tropical diseases (NTDs) that can be controlled with ivermectin such as lice, scabies, cutaneous larval migrans (CLM) [43], the soil-transmitted helminths (STHs) (e.g., *Ascaris*, *Trichuris*, and hookworm) [44, 45], and strongyloidiasis [46]. The OEPA once yearly ivermectin MDA reduced *Strongyloides* and *Trichuris* prevalence, but not *Ascaris* or hookworm in Ecuador [47] and Colombia [48]. Indeed, ivermectin can be quite effective against lice, scabies, CLM, STHs and strongyloidiasis when administered once [49] or twice within 7–10 days [50–52] and MDAs can be quite effective [51, 52]. However, re-infection from the soil for CLM, STHs and *Strongyloides*, and re-infestation from untreated persons for scabies and lice can occur quickly [52]. This suggests that more frequent ivermectin MDAs to target malaria parasite transmission could have dramatic impact on numerous NTDs found in Latin America, and may improve compliance with MDAs for malaria control.

*Plasmodium vivax* has become the most prevalent malaria species in Latin America. Due to the relapsing nature and inability to identify persons with *P. vivax* hypnozoites, this species will be considerably more difficult to eliminate. Several field trials are under way to eliminate *P. falciparum* by administering MDAs with dihydroartemisinin–piperaquine and low-dose primaquine [53, 54]. It has been observed that persons infected with *P. falciparum* also have dormant *P. vivax* hypnozoites that release after *P. falciparum* treatment [55–58]. Dihydroartemisinin–piperaquine is effective against blood stage *P. vivax* and the long half-life of piperaquine with once a month administrations can effectively suppress the frequent tropical *P. vivax* relapses at the blood stage [59]. Primaquine MDAs with the target of radical cure of *P. vivax* hypnozoites have been performed in several countries including: Afghanistan, Azerbaijan, Tajikistan, North Korea [60], Taiwan, Papua New Guinea, Solomon Islands, Tanzania, Nicaragua, Malaysia, Indonesia, China, Kyrgyzstan [61], Vanuatu [62], and Cambodia [63]. Since the total cumulative dose of primaquine provides radical cure of *P. vivax* hypnozoites [64] it is possible to space the primaquine MDAs every 7 [62] to 10 [63] days over several months. Ivermectin could be co-administered with primaquine MDAs every 7–10 days to achieve substantial suppression by *Anopheles* vectors for all *Plasmodium* species while specifically targeting *P. vivax* radical cure. Currently, clinical trials to investigate the safety and tolerability of ivermectin plus dihydroartemisinin–piperaquine (NCT02568098) [65] and ivermectin plus primaquine (NCT02568098) are being conducted. If ivermectin can be safely co-administered with anti-malarial drugs during MDAs, then this has the potential to be a powerful malaria and vector control intervention in Latin America.

## Conclusions

Ivermectin reduces *An. darlingi* survivorship, modestly inhibits development of *P. vivax* in the vector by reducing oocyst prevalence at the LC_50_ and LC_25_ but not intensity, and delays time to re-feed at human-relevant concentrations up to 12 h post drug ingestion. The ivermectin 400-μg/kg dose is likely the ideal minimal dose used during ivermectin MDA in Latin America. The success of the OEPA against onchocerciasis indicates that ivermectin MDAs can be effectively executed in Latin America. Numerous NTDs that are prevalent in Latin America could be affected by ivermectin MDAs. This suggests that ivermectin MDAs could be a powerful new tool to aid malaria elimination in Latin America and would likely be well received as a public health measure.

## Abbreviations

CLMs: cutaneous larval migrans
DMSO: dimethylsulfoxide
MDA: mass drug administration
NAMRU-6: Naval Medical Research Unit No. 6
NTDs: neglected tropical diseases
OEPA: Onchocerciasis Elimination Programme for the Americas
PBS: phosphate-buffered saline
STHs: soil-transmitted helminths
